# Knockdown of *miR-128a* induces *Lin28a* expression and reverts myeloid differentiation blockage in acute myeloid leukemia

**DOI:** 10.1038/cddis.2017.253

**Published:** 2017-06-01

**Authors:** Luciana De Luca, Stefania Trino, Ilaria Laurenzana, Daniela Tagliaferri, Geppino Falco, Vitina Grieco, Gabriella Bianchino, Filomena Nozza, Valentina Campia, Francesca D'Alessio, Francesco La Rocca, Antonella Caivano, Oreste Villani, Daniela Cilloni, Pellegrino Musto, Luigi Del Vecchio

**Affiliations:** 1 Laboratory of Preclinical and Translational Research, IRCCS – Referral Cancer Center of Basilicata (CROB), Rionero in Vulture 85028, Italy; 2 Biogem Scarl, Istituto di Ricerche Genetiche ‘Gaetano Salvatore’, Ariano Irpino 83031, Italy; 3 Laboratory of Clinical Research and Advanced Diagnostics, IRCCS – Referral Cancer Center of Basilicata (CROB), Rionero in Vulture 85028, Italy; 4 Department of Clinical and Biological Sciences, University of Turin, Orbassano 10043, Italy; 5 CEINGE Biotecnologie Avanzate scarl, Naples 80147, Italy; 6 Department of Onco-Hematology, IRCCS – Referral Cancer Center of Basilicata (CROB), Rionero in Vulture 85028, Italy; 7 Scientific Direction, IRCCS – Referral Cancer Center of Basilicata (CROB), Rionero in Vulture 85028, Italy; 8 Department of Molecular Medicine and Medical Biotechnologies, University of Naples Federico II, Napoli 80138, Italy

## Abstract

Lin28A is a highly conserved RNA-binding protein that concurs to control the balance between stemness and differentiation in several tissue lineages. Here, we report the role of *miR-128a/Lin28A* axis in blocking cell differentiation in acute myeloid leukemia (AML), a genetically heterogeneous disease characterized by abnormally controlled proliferation of myeloid progenitor cells accompanied by partial or total inability to undergo terminal differentiation. First, we found Lin28A underexpressed in blast cells from AML patients and AML cell lines as compared with CD34+ normal precursors. *In vitro* transfection of Lin28A in NPM1-mutated OCI-AML3 cell line significantly triggered cell-cycle arrest and myeloid differentiation, with increased expression of macrophage associate genes (*EGR2*, *ZFP36* and *ANXA1*). Furthermore, *miR-128a*, a negative regulator of *Lin28A*, was found overexpressed in AML cells compared with normal precursors, especially in acute promyelocytic leukemia (APL) and in ‘AML with maturation’ (according to 2016 WHO classification of myeloid neoplasms and acute leukemia). Its forced overexpression by lentiviral infection in OCI-AML3 downregulated Lin28A with ensuing repression of macrophage-oriented differentiation. Finally, knockdown of *miR-128a* in OCI-AML3 and in APL/AML leukemic cells (by transfection and lentiviral infection, respectively) induced myeloid cell differentiation and increased expression of *Lin28A*, *EGR2*, *ZFP36* and *ANXA1*, reverting myeloid differentiation blockage. In conclusion, our findings revealed a new mechanism for AML differentiation blockage, suggesting new strategies for AML therapy based upon *miR-128a* inhibition.

Acute myeloid leukemia (AML) is a heterogeneous hematopoietic stem cell neoplasm, characterized by rapid growth and/or impaired differentiation of leukemic cells with abnormal accumulation.^1, 2, 3^ Recurring chromosomal aberrations and gene mutations contribute to AML pathogenesis and are the most important tools for classification and prognosis assessment of AML.^2, 3, 4^ Furthermore, there are some known deregulated pathways involved in the maintenance of leukemic stem cells such as hedgehog,^5, 6^ tyrosine kinase receptors (e.g. Flt3),^3, 7^ Wnt and Notch.^8, 9, 10, 11^ Notwithstanding, a successful target therapy is not yet available. Improving our current knowledge on the biology of AML-associated leukemic processes represents a valuable tool to identify novel potential drug targets.

Lin28 is a conserved RNA-binding protein having an important role in cancer stem cells.^12, 13^ This protein is expressed in embryonic stem cells^14, 15^ and is capable, with OCT4, SOX2 and NANOG, of converting fibroblasts in induced pluripotent stem cells.^16^ Lin28, by physical interaction with several RNA transcripts, exerts various forms of regulation ranging from alternative splicing, turnover, localization and translation.^17, 18, 19^ It has been demonstrated that altered functionality of RNA-binding proteins, due to deregulated gene expression or gene mutations, often results in genetic disease and cancer.^20^

Several studies reported the existence of regulatory pathways between Lin28 and different miRNAs.^15, 21, 22, 23^ In murine model, overexpression of *miR-125b* leads to the downregulation of Lin28A and the preleukemic state characterized by overproduction of myeloid cells eventually progressing to a myeloid leukemia.^24, 25, 26^ Conversely, ectopic expression of Lin28B reprograms hematopoietic progenitor cells from adult bone marrow (BM), endowing them to mediate multilineage reconstitution.^27^ Moreover, Li *et al.*^22^ showed that *miR-181* promotes megakaryocytic differentiation repressing Lin28 and upregulating let-7 expression. Thus, Lin28 seems to be a pivotal regulator of hematopoiesis. Interestingly, Lin28 is also regulated by *miR-128*,^28^ a microRNA able to hold hematopoietic cells in an early progenitor stage, blocking their differentiation towards more mature cells.^29, 30^ Moreover, this microRNA was found associated with AML.^31, 32, 33^ Therefore, it will be appealing to gain further insights into the role of *miR-128a/Lin28A* axis in induction and maintenance of an early differentiation status in AML.

## Results

### *Lin28A* expression was downregulated in myeloid leukemic cells

**Figure 1:**
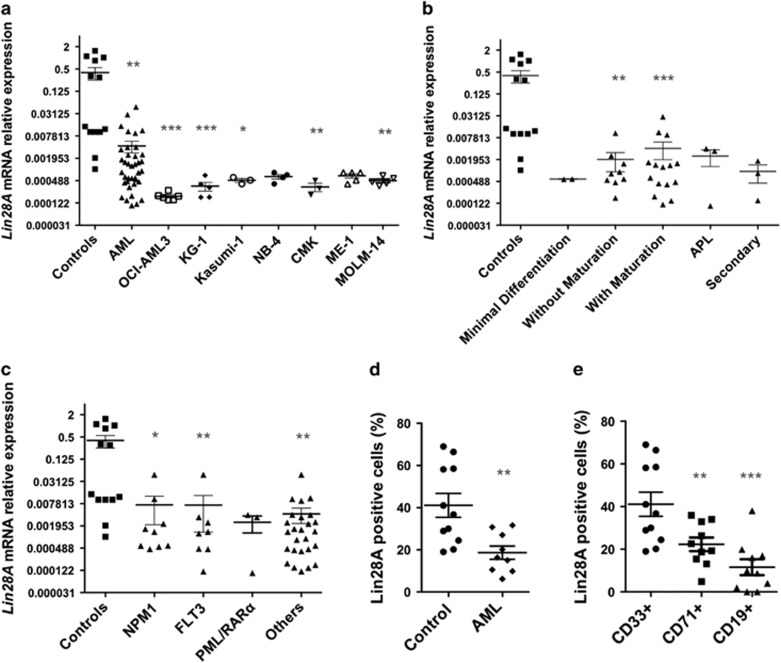
*Lin28A* expression in leukemic blasts from AML patients. (**a**) qRT-PCR of *Lin28A* in 13 healthy controls, 38 AML patients and 7 AML cell lines (OCI-AML3, KG-1, Kasumi-1, NB4, CMK, ME-1 and MOLM-14); *ABL1* was used for normalization. Relative values were calculated on the basis of the ΔCp method. Results are shown as mean±S.E.M. (**b**) Expression of *Lin28A* mRNA in AML patients stratified for morphologic features (with minimal differentiation, *n*=2; without maturation, *n*=9; with maturation including: *n*=3 with maturation, *n*=10 acute myelomonocytic leukemia, *n*=2 acute monoblastic/monocytic leukemia; APL, *n*=3; secondary AML, *n*=3) was compared with 13 healthy controls. Results are shown as mean±S.E.M. (**c**) Expression of *Lin28A* mRNA in AML patients with specific mutations (NPM1, *n*=9; FLT3, *n*=8; PML/RAR*α*, *n*=3 or with other alterations, *n*=26) was compared with 13 healthy controls. Results are shown as mean±S.E.M. (**d**) Percentage of Lin28A+ cells in 11 BM healthy controls and 9 AML patients, by cytofluorimetric analysis. (**e**) Percentage of Lin28A+ cells in normal myeloid (CD34+ CD45+ CD33+), erythroid (CD34+ CD45+ CD71+) and lymphoid (CD34+ CD45+ CD19+) precursors, by cytofluorimetric analysis. Statistically significant analyses are indicated by asterisks: *P<0.05, **P<0.01 and ***P<0.001

To evaluate *Lin28A* expression in AML, we performed quantitative real-time-PCR (qRT-PCR) in isolated blast cell samples from 38 AML patients at diagnosis, 7 AML cell lines (OCI-AML3, KG-1, Kasumi-1, NB4, CMK, ME-1 and MOLM-14) and CD34+ purified samples from 13 healthy donors. *Lin28A* (*P*<0.01) and cell lines (OCI-AML3 and KG-1 *P*<0.001, Kasumi-1, NB4, CMK and ME-1, *P*<0.01) showed a significantly lower expression in AML patients as compared with controls (Figure 1a). To support our data, we also analyzed two independent publicly available gene expression profiling data sets, one containing 16 CD34+ isolated samples from healthy subjects (GSE 42519), and one with 251 AML patients with newly diagnosed AML (GSE 15434) confirming a significant downregulation of *Lin28A* in AML patients (230 BM and 21 PB) compared with healthy subjects (Supplementary Figure 1a). Stratifying AML according to the WHO classification,^4^ *Lin28A* value was found underexpressed in all AML subtypes (Figure 1b) compared with controls. Stratifying AML cases according to the principal genomic alterations detected in our cohort of patients and in GSE 15434 data set, we found lower expression of Lin28A in AML patients independent of their specific alterations (Figure 1c and Supplementary Figure 1b). Moreover, we evaluated Lin28A protein by cytometric analysis detecting a lower percentage of Lin28A+ cells in AML blast cells compared with normal hematopoietic myeloid precursors (*P*<0.01) (Figure 1d). When we analyzed distinct subsets of normal CD34+ cells, we observed a higher percentage of Lin28A+ cells in normal myeloid precursors (CD33+) compared with the erythroid (CD71+) (*P*<0.01) and lymphoid (CD19+) (*P*<0.001) ones, suggesting its main involvement in myeloid differentiation (Figure 1e).

### Lin28A overexpression induced hematopoietic differentiation in AML

**Figure 2:**
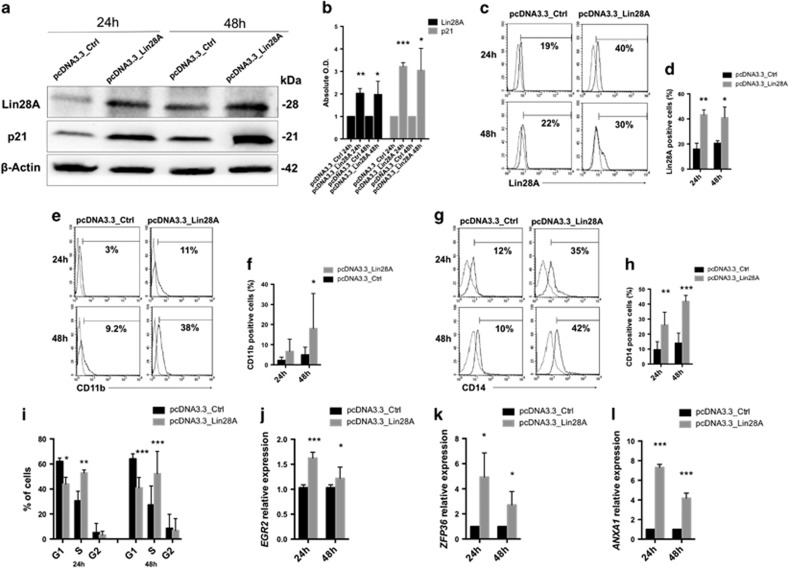
Overexpression of Lin28A in OCI-AML3 cell line. (**a**) Western blotting (WB) analysis of Lin28A, p21 and *β*-actin in OCI-AML3 after 24 and 48 h of transfection with pcDNA3.3_Ctrl or pcDNA3.3_Lin28A plasmids. (**b**) Absolute OD values of (**a**) were normalized to *β*-actin and shown as mean±S.D. from two independent experiments. (**c**) Representative cytofluorimetric analysis of percentage Lin28A+ cells in OCI-AML3 after 24 and 48 h of transfection with pcDNA3.3_Ctrl or pcDNA3.3_Lin28A plasmids. (**d**) Percentage of Lin28A+ OCI-AML3 cells after 24 and 48 h of transfection with pcDNA3.3_Ctrl or pcDNA3.3_Lin28A plasmids, by cytofluorimetric analysis. (**e**) Representative cytofluorimetric analysis of CD11b+ cells in OCI-AML3 after 24 and 48 h of transfection with pcDNA3.3_Ctrl or pcDNA3.3_Lin28A plasmids. (**f**) Percentage of CD11b+ OCI-AML3 cells after 24 and 48 h of transfection with pcDNA3.3_Ctrl or pcDNA3.3_Lin28A plasmids. (**g**) Representative cytofluorimetric analysis of CD14+ cells in OCI-AML3 after 24 and 48 h of transfection with pcDNA3.3_Ctrl or pcDNA3.3_Lin28A plasmids. (**h**) Percentage of CD14+ OCI-AML3 cells after 24 and 48 h of transfection with pcDNA3.3_Ctrl or pcDNA3.3_Lin28A plasmids, by cytofluorimetric analysis. (**i**) Cell-cycle analysis in OCI-AML3 after 24 and 48 h of transfection with pcDNA3.3_Ctrl or pcDNA3.3_Lin28A plasmids. (**j–l**) qRT-PCR of *EGR2* (**j**) *ZFP36* (**k**) and *ANXA1* (**l**) in OCI-AML3 after 24 and 48 h of transfection with pcDNA3.3_Ctrl or pcDNA3.3_Lin28A plasmids. The bar graphs represented mean±S.D. from three independent experiments. Statistically significant analyses are indicated by asterisks: *P<0.05, **P<0.01 and ***P<0.001

To examine the effect of Lin28A in AML, we transfected OCI-AML3 cells with Lin28A plasmid. The significant increase of Lin28A protein expression was confirmed by western blot and cytofluorimetric analysis (*P*<0.01 at 24 h and *P*<0.05 at 48 h, in both cases) (Figures 2a–d). Lin28A overexpression was associated with the induction of monocyte/macrophage-like differentiation. In fact, flow cytometric analysis revealed a higher percentage of CD11b− (*P*<0.05 at 48 h) and CD14+ cells (*P*<0.01 at 24 h and *P*<0.001 at 48 h) after Lin28A transfection (Figures 2e–h). Ectopic expression of Lin28A also significantly increased p21 protein levels (*P*<0.001 at 24 h and *P*<0.05 at 48 h), inducing cell-cycle arrest in the S phase (*P*<0.01 at 24 h and *P*<0.001 at 48 h) (Figures 2a and i). Consistent to the ability of Lin28A in inducing hematopoietic differentiation in AML cells, we detected a significant increase of *EGR2* and *ZFP36*, two key regulators of monocyte/macrophage differentiation (Figures 2j–k),^34, 35, 36^ and *ANXA1*, a gene normally stored in inside macrophage cytosol (Figure 2l)^37^ after Lin28A overexpression at 24–48 h.

### *Lin28A* expression increased during PMA or ATRA differentiation

**Figure 3:**
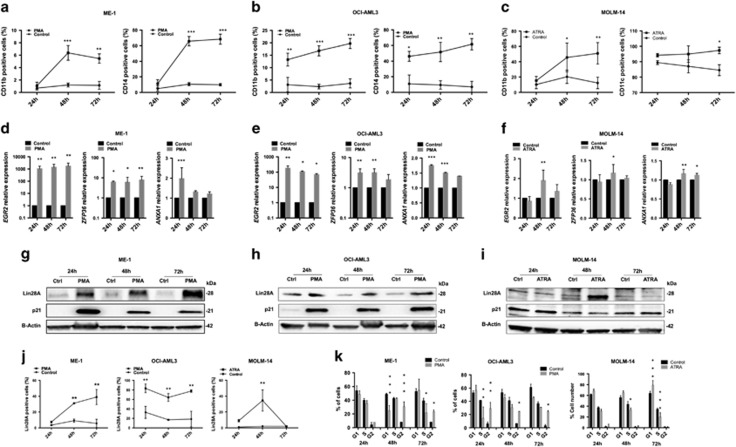
Lin28A upregulation during macrophage-like differentiation in AML cell lines. (**a** and **b**) Percentage of CD11b+ and CD14+ cells in ME-1 (**a**) and OCI-AML3 (**b**) after 24, 48 and 72 h of treatment with PMA, by cytofluorimetric analysis. (**c**) Percentage of CD11b+ and CD11c+ cells of MOLM-14 after 24, 48 and 72 h of treatment with ATRA, by cytofluorimetric analysis. (**d–f**) qRT-PCR of *EGR2*, *ZFP36* and *ANXA1* in ME-1 (**d**), OCI-AML3 (**e**) and MOLM-14 (**f**) after 24, 48 and 72 h of treatment with PMA or ATRA. (**g–i**) Western blotting (WB) analysis of Lin28A, p21 and *β*-actin in ME-1 (**g**), OCI-AML3 (**h**) and MOLM-14 (**i**) after 24, 48 and 72 h of treatment with PMA. (**j**) Percentage of Lin28A+ ME-1, OCI-AML3 and MOLM-14 cells after 24, 48 and 72 h of treatment with PMA or ATRA, by cytofluorimetric analysis. (**k**) Cell-cycle analysis in ME-1, OCI-AML3 and MOLM-14 cells after 24, 48 and 72 h of treatment with PMA or ATRA. The line and bar graphs represented mean±S.D. from three independent experiments. Statistically significant analyses are indicated by asterisks: *P<0.05, **P<0.01 and ***P<0.001

To corroborate the involvement of Lin28A in myeloid differentiation, we stimulate AML cell lines to differentiate. In particular, we induced macrophage-like differentiation treating ME-1/OCI-AML3 cell lines with phorbol 12-myristate 13-acetate (PMA) and MOLM-14 with all-*trans*-retinoic acid (ATRA), and granulocyte-like differentiation treating NB4 and KG-1 cell lines with ATRA. After treatment, the cytometric data revealed a significant percent increase, from 24 to 72 h, of CD11b+ cells and CD14+ cells in ME-1, OCI-AML3 (Figures 3a and b) and NB4 (Supplementary Figure 2a), of CD11b and CD11c in MOLM-14 (Figure 3c) and of CD11b and CD15 in KG-1 (Supplementary Figure 2b). To confirm cytometric analysis of cell differentiation, we detected by qRT-PCR a significant augment, in all time points, of *EGR2*, *ZFP36* and *ANXA1* in treated ME-1, OCI-AML3, MOLM-14 (Figures 3d–f) and NB4 (Supplementary Figure 2c).^37^ As expected, at the same time, we observed a significant upregulation of Lin28A and an increased percentage of Lin28A+ cells in all cell lines (Figures 3g–j and Supplementary Figures 2d and e). Similarly to Lin28A transfection, PMA and ATRA treatment of AML cell lines also induced p21 expression (Figures 3g–i) and a significant cell-cycle arrest in the G2 phase (ME-1: *P*<0.001 at 48 h, *P*<0.05 at 72 h; OCI-AML3: *P*<0.01 at 24 h, *P*<0.05 at 48 and 72 h; KG-1: *P*<0.001 at 24 h, *P*<0.05 at 48 h, *P*<0.001 at 72 h) (Figure 3k and Supplementary Figure 2f), the G1 phase (MOLM-14: *P*<0.001 at 72 h) (Figure 3k) or the S phase (NB4: *P*<0.001 at 24 h, *P*<0.01 at 48 and 72 h) (Supplementary Figure 2f).

### *MiR-128a* expression was upregulated in myeloid leukemic cells

**Figure 4:**
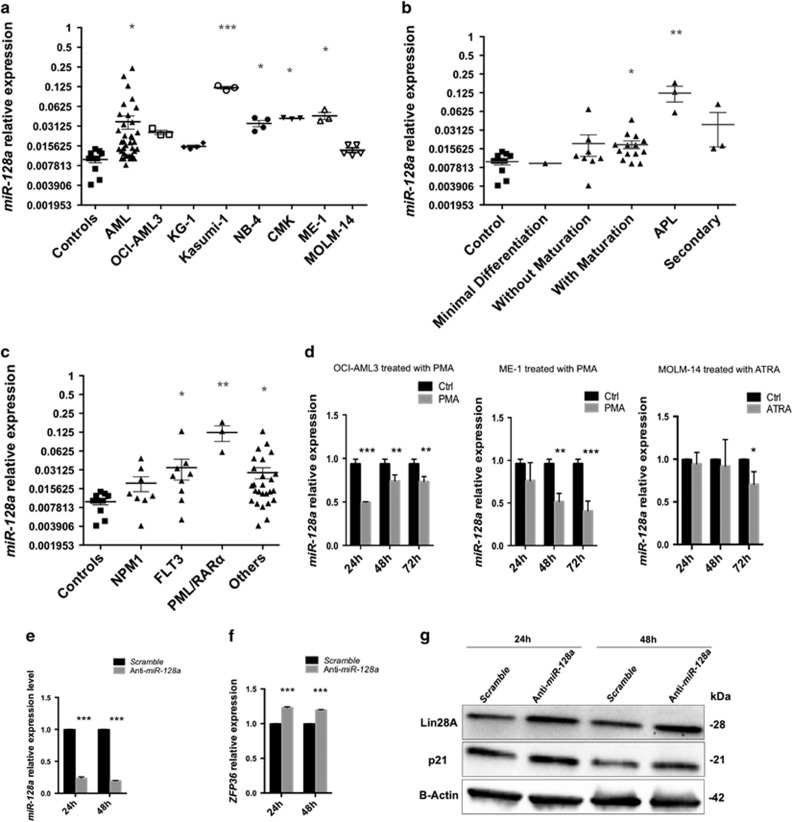
*MiR-128a* expression in leukemic blasts from AML patients and its inhibition in OCI-AML3 cell line. (**a**) qRT-PCR of *MiR-128a* in 10 healthy controls, 35 AML patients and 6 AML cell lines (OCI-AML3, KG-1, Kasumi-1, NB4, CMK, ME-1 and MOLM-14); *RNU44* was used for normalization. Relative values were calculated on the basis of the ΔCp method. Results are shown as mean±S.E.M. (**b**) Expression of *miR-128a* in AML patients stratified for morphologic features (with minimal differentiation, *n*=1; without maturation, *n*=8; with maturation including: *n*=3 with maturation, *n*=9 acute myelomonocytic leukemia, *n*=2 acute monoblastic/monocytic leukemia; APL, *n*=3; secondary AML, *n*=3) was compared with 10 healthy controls. Results are shown as mean±S.E.M. (**c**) Expression of *miR-128a* in AML patients with specific mutations (NPM1, *n*=8, FLT3, *n*=9 or with other alterations, *n*=26) was compared with 10 healthy controls. Results are shown as mean±S.E.M. (**d**) qRT-PCR of *miR-128a* in OCI-AML3, ME-1 and MOLM-14 cells after 24, 48 and 72 h of treatment with PMA or ATRA. The bar graphs represented mean±S.D. from three independent experiments. (**e** and **f**) qRT-PCR of *miR-128a* (**e**) and *ZFP36* (**f**) in OCI-AML3 after 24 and 48 h of scramble or anti-*miR-128a* transfection. The bar graphs represented mean±S.D. from three independent experiments. (**g**) Western blotting (WB) analysis of Lin28A, p21 and *β*-actin in OCI-AML3 after 24 and 48 h of transfection with scramble or anti-*miR-128a*. Statistically significant analyses are indicated by asterisks: *P<0.05, **P<0.01 and ***P<0.001

To further clarify Lin28A downregulation in AML, we analyzed its regulator, *miR-128a*.^28^ We evaluated *miR-128a* expression in the same cohort of AML patients and in the AML cell line panel previously examined for *Lin28A*, observing a significant overexpression of this microRNA compared with healthy subjects (Figure 4a). Stratifying AML cases for morphologic features, we found, at variance with Lin28A, elevated expression levels of *miR-128a* in AML with maturation and acute promyelocytic leukemia (APL) cases compared with controls (Figure 4b). Furthermore, considering patients for their gene mutations, we found a significantly higher expression of *miR-128a* in patients with FLT3, PML/RAR*α* and other genomic alterations (Figure 4c).

Our results show different expression pattern of *miR-128a* in MOLM-14 and AML samples, both carrying FLT3-ITD (Figures 4a and c). Matsuo *et al.*^38^ demonstrated that MOLM-14, along with FLT3-ITD, carries a series of genotypic aberrancies, such as the insertion ins(11;9) with the fusion hybrid MLL-AF9.^38^ This complex pattern could justify the partially divergent behavior of MOLM-14 as compared with fresh AML samples. Moreover, we also evaluated, by qRT-PCR, *miR-128a* expression during macrophage- and granulocytic-like differentiation detecting a significant downregulation of this *microRNA* from 24 to 72 h in treated cell lines (Figure 4d and Supplementary Figure 3a). To determine the role of *miR-128a* in myeloid differentiation, we transiently transfected OCI-AML3 and ME-1 cells with anti*-miR-128a.* After transfection, the inhibition of *miR-128a* (Figure 4e and Supplementary Figure 4a) and the increase of *ZFP36* were confirmed by qRT-PCR assay (Figure 4f and Supplementary Figure 4b), thus supporting a role of *miR-128a* in monocyte/macrophage differentiation. Furthermore, to confirm a *miR-128/Lin28A* axis, we evaluated *Lin28A* expression after anti-*miR-128a* transfection, confirming its upregulation in both cell lines (Figure 4g and Supplementary Figure 4c); we also observed an increase of p21 in OCI-AML3 cells (Figure 4g).

### *MiR-128a* overexpression altered macrophage- and granulocytic-like differentiation

**Figure 5:**
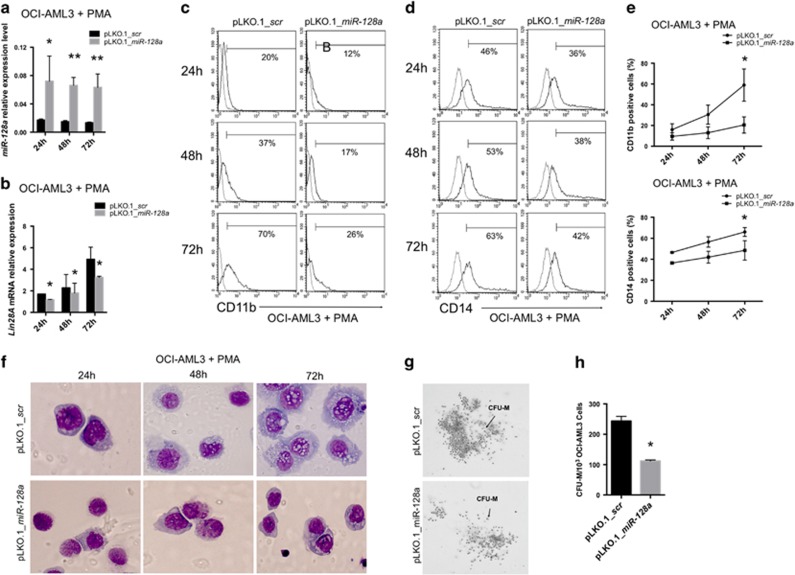
*MiR-128a* overexpression in OCI-AML3 cell line. (**a** and **b**) qRT-PCR of *miR-128a* (**a**) and *Lin28A* (**b**) in OCI-AML3 infected with pLKO.1_scr or pLKO.1_miR-128a after 24, 48 and 72 h of PMA treatment. (**c** and **d**) Representative histogram plots of CD11b+ (**c**) and CD14+ cells (**d**) in OCI-AML3 infected with pLKO.1_scr or pLKO.1_miR-128a after 24, 48 and 72 h of PMA treatment. (**e**) Percentage of CD11b+ and CD14+ OCI-AML3 cells infected with pLKO.1_scr or pLKO.1_miR-128a after 24, 48 and 72 h of PMA treatment, by cytofluorimetric analysis. (**f**) May–Grünwald Giemsa staining of OCI-AML3 infected with pLKO.1_scr or pLKO.1_miR-128a after 24, 48 and 72 h of PMA treatment. (**g**) Colony-forming assay of OCI-AML3 after infection with pLKO.1_scr or pLKO.1_miR-128a. Colonies were observed at day 14 of the semisolid culture under × 20 magnification. (**h**) Count of CFU-M colonies. The line and bar graphs represented mean±S.D. from three independent experiments. Statistically significant analyses are indicated by asterisks: *P<0.05 and **P<0.01

To examine the effect of *miR-128a* in AML, we overexpressed by lentiviral infection its microRNA precursor (pLKO.1_*miR-128a*) and, as a control, an empty vector (pLKO.1_*scr*) in OCI-AML3 (Supplementary Figure 5). After lentivirus infection, cells were treated with PMA to differentiate in macrophage-like cells. Although during differentiation *miR-128a* expression seemed to be reduced in treated cells, its levels remained significantly higher in pLKO.1_*miR-128a* cells than in pLKO.1_*scr* cells (*P*<0.05 at 24 h, *P*<0.01 at 48 h and 72 h) (Figure 5a). Concurrently, *Lin28A* expression increased as a consequence of the induction culture, but it was significantly downregulated in OCI-AML3 infected with *miR-128a* (*P*<0.05 at 24–72 h) compared with control (Figure 5b).

Overexpression of *miR-128a* inhibited macrophage-like differentiation markers. In fact, flow cytofluorimetric data showed a reduction of CD11b+ and CD14+ cells after 24, 48 and 72 h of treatment with PMA (Figures 5c–e) in pLKO.1_*miR-128a* cells compared with that in pLKO.1_*scr* cells (*P*<0.05 at 72 h). These data were confirmed by morphologic analysis with May–Grünwald Giemsa staining of infected cells, highlighting that *miR-128a* overexpression led to less mature macrophage-like cells (Figure 5f). Moreover, lentiviral infection of *miR-128a* inhibited colony-forming activity of colony-forming unit-macrophage (CFU-M) in colony size and number (Figures 5g and h).

### Inhibition of *miR-128a* improved myeloid differentiation in AML BM HSPC

**Figure 6:**
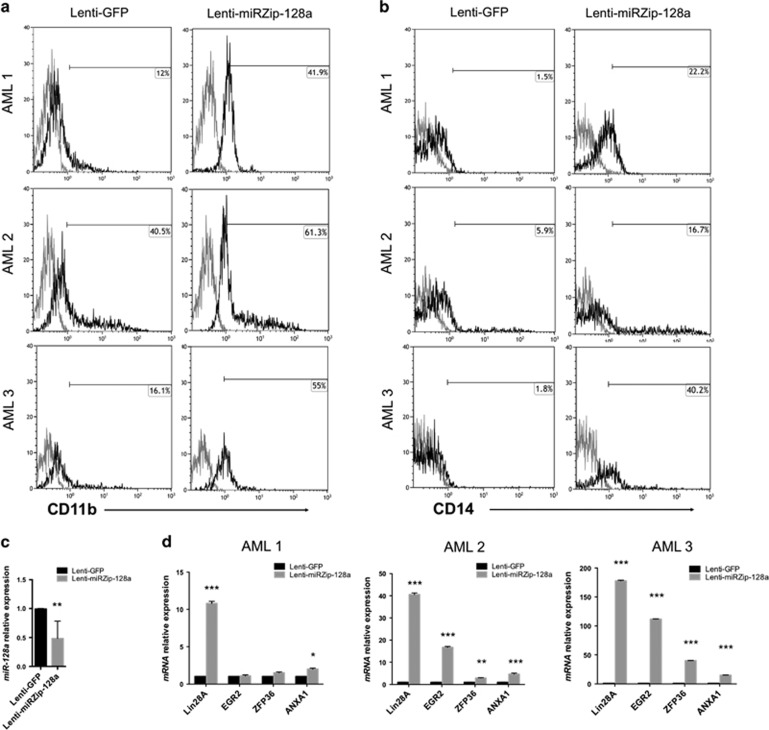
Inhibition of *miR-128a* in leukemic cells from AML patients. (**a** and **b**) Percentage of CD11b+ (**a**) and CD14+ cells (**b**) from three AML patients (AML1, AML2 and AML3) infected with Lenti-GFP or Lenti-miRZip-128a after 3 days of macrophage-like induction culture, by cytofluorimetric analysis. (**c**) qRT-PCR of *miR-128a* in AML patients infected with Lenti-GFP or Lenti-miRZip-128a. (**d**) qRT-PCR of *Lin28A*, *EGR2*, *ZFP36* and *ANXA1* in AML patients infected with Lenti-GFP or Lenti-miRZip-128a after 3 days of macrophage-like induction culture. Statistically significant analyses are indicated by asterisks: *P<0.05, **P<0.01 and ***P<0.001

Since significantly increased *miR-128a* expression was mainly observed in AML with maturation, we investigated how *miR-128a* inhibition could influence myeloid differentiation/maturation blockage. Lenti-miRZip-128a stably expresses hairpins that have anti-miRNA activity. We used BM HSPCs derived from two AML patients with maturation (myeloblastic AML3 and myelomonocyte AML2, respectively), both FLT3 mutated, and one APL patient (AML1) (Supplementary Table S1). BM HSPCs were infected with Lenti-miRZip-128a or Lenti-GFP and exposed to macrophage-like induction culture. Flow cytometric analysis showed a significant increased of CD11b and CD14 percentage of positive cells in AML HSPCs infected with Lenti-miRZip-128a compared with the control (Figures 6a and b). Lenti-miRZip-128a infection decreased the levels of mature *miR-128a* (Figure 6c) and significantly enhanced the expression of *Lin28A*, *EGR2*, *ZFP36* and *ANXA1* (Figure 6d). These results demonstrated that *miR-128a* inhibition in AML induce myeloid differentiation.

## Discussion

AMLs are clonal diseases of hematopoietic progenitor cells, characterized by marked heterogeneity in terms of phenotypic, genotypic and clinical features.^1, 2, 4, 39^ In this study, we showed that *Lin28A*, an RNA-binding protein,^12^ was significantly underexpressed in AML samples without any association with genotypic and phenotypic stratification. Moreover, we found a higher percentage of Lin28A+ cells in myeloid precursors compared with that in erythroid and lymphoid normal precursors, suggesting a preferential involvement of this protein in myeloid lineage differentiation.

Recently, Chaudhuri *et al.*^26^ demonstrated that the knockdown of Lin28A in mouse hematopoietic system led to myeloid cell expansion and decrease of B-cell number, thus triggering an alteration of hematopoiesis. Furthermore, its overexpression in normal HSC produced a significant reduction of total white blood cells, causing mice dead at 5 weeks, probably because of the impaired hematopoietic development.^26^

Our data, instead, showed that Lin28A overexpression in AML cells activated myeloid maturation. We observed, in fact, an increase of myeloid differentiation markers and a cell-cycle arrest with p21 expression augment. Literature data demonstrated that p21, a cyclin-dependent kinase inhibitor, induced cell-cycle arrest if overexpressed in progenitor cells favoring macrophage differentiation because of the accumulation of PU.1, a lineage-determining factor.^40^ Of importance, we also detected a significant increase of macrophage-specific genes like *early growth response 2* (*EGR2*), an EGR protein involved in macrophage growth and differentiation,^34, 41^ tristetraprolin (*ZFP36*), an anti-inflammatory and anticarcinogenic protein that is also involved in monocyte/macrophage differentiation processes and *annexin A1* (*ANXA1*) an anti-inflammatory protein stored in the macrophage cytosol.^37, 42^ In addition, we demonstrated that Lin28A is a positive regulator of granulocytic- and macrophage-like differentiation. In fact, we observed its significant increase simultaneously augmented different myeloid-specific markers, stimulated by ATRA or PMA treatment, in five AML cell lines with different genotype and morphology.

Previous studies reported that Lin28A is a direct target of *miR-128*,^28^ a microRNA involved in hematopoiesis.^29, 30^ Different studies have associated *miR-128a* with leukemia, showing that *miR-128a* belongs to a set of miRNAs with stringent specificity for AML or ALL.^31, 32, 33^ Moreover, *miR-128a* expression was found to be associated with a subgroup of AML patients with high-risk molecular features, refractoriness, relapse and death.^31, 33^

In our study, we evaluated *miR-128a* expression in our cohort of AML patients. Of interest, *miR-128a* showed a significantly higher level in APL and AML with mature phenotypes harboring *FLT3* and/or other alterations. Qian *et al.*^28^ sustained that *miR-128* directly target *BMI1, CSF1, KLF4, LIN28A, NANOG* and *SNAIL*. Some of these genes are involved in self-renewal (*Bmi1* and *Nanog*)^43^ and differentiation (*CSF1* and *KLF4*).^44, 45^ Similar to Lin28A, they are deregulated in AML.^45, 46, 47^ KLF4, for example, a lineage-specific transcriptor factor that promotes monocyte differentiation is downregulated in undifferentiated subtype M0 and in FLT3-ITD and NPM1-mutant AML.^45^ BMI1, instead, a polycomb group protein involved in self-renewal is overexpressed in different AML subtypes.^46^ Given that gene regulation is complex and depend on different factors,^45, 48, 49, 50^ the relative upregulation of *miR-128* could not be sufficient to repress all these genes.

Various microRNAs have an important role in acute myeloid leukemogenesis,^50, 51^ because of their role in the different stages of hematopoiesis.^29, 52^ *MiR-125b*, for example, is overexpressed in certain types of AML (C/EBP*α*, t(2;11)(p21;q23), GATA1) and inhibits myeloid differentiation.^50, 53^ Moreover, its overexpression causes a dose-dependent myeloproliferative disorder progressing to a lethal myeloid leukemia in mice.^50^ *MiR-181* family, instead, was found abnormally upregulated in AML patients, with t(8;21) and t(15;17) inhibiting granulocytic- and macrophage-like differentiations.^54^ Here, we demonstrated that *miR-128a* was downregulated during induced granulocyte- and macrophage-like differentiation of AML cell lines. Moreover, we showed a reduction of Lin28A- and myeloid-specific marker expression following enforced *miR-128a* expression, in spite of PMA treatment *in vitro*. Conversely, *miR-128a* transient inhibition in two cell lines enhanced myeloid maturation and Lin28A overexpression. Given the higher expression of *miR-128a* in AML with mature phenotypes and with *FLT3* or *PML/RARα* alterations, we decided to inhibit *miR-128a* maturation in leukemic cells of these subsets of patients to stimulate further propensity to cell differentiation. In fact, Lenti-miRZip-128a infection remarkably repressed *miR-128a* and improved granulocytic/macrophage-like differentiation in BM-derived AML blasts. Finally, we detected an augment of *Lin28A* in all infected AML blasts patients, while an increase of macrophage-specific genes occurred only in AML with *FLT3* mutation and mature phenotypes.

Specific microRNAs with established oncogenic functions, such as *miR-155*, *miR-125b*, *miR-181* and *miR-128a*, appear to be associated with particular AML subtypes.^31, 50, 55^ Selected sets of microRNAs could be used as a target therapy tailored to specific biological and molecular features of AML.^50^ In particular, we hypothesize that in AML subtypes with t(8;21) and inv16, differentiation block could be released by *miR-128a* knockdown in combination with differentiation agents. In this setting, we previously demonstrated that G-CSF treatment of a patient with t(8;21) AML led to complete remission.^56^ Moreover, the combined inhibition of *miR-128a* and *miR-155* could be evaluated as a therapeutic option in high -isk AML patients harboring *FLT3* mutation.

In conclusion, we revealed a new regulatory axis *miR-128a/Lin28A* that affects hematopoiesis, favoring AML development. Our experiments suggest that the inhibition of *miR-128a* could provide a new strategy for AML therapy.

## Materials and methods

### Human samples

BM samples were obtained from 40 AML patients (37 *de novo* and 3 secondary AML) at the time of diagnosis from the IRCCS CROB Hospital. The clinical and biological characteristics of AML patients are summarized in Supplementary Table S1. All patients gave written informed consent according to the Declaration of Helsinki. BM and peripheral blood samples of 13 healthy donors were also obtained from San Luigi Gonzaga Hospital of Turin. CD34+ cells of all samples were purified from mononuclear cells with a CD34 Microbead Kit (Miltenyi Biotec, Auburn, CA, USA). The purity of immunoselected cells routinely ranged between 90 and 95% and it was assessed by flow cytometric analysis using an allophycocyanin (APC) anti-CD34 (BD Pharmingen, San Jose, CA, USA).

### Cell lines

The human AML cell lines, OCI-AML3, KG-1, Kasumi-1, NB4, CMK, ME-1 and MOLM-14, were acquired from American Type Culture Collection (Rockville, MD, USA) or Deutsche Sammlung von Mikroorganismen und Zellkulturen (Braunschweig, Germany). AML cell line characteristics were reported in Supplementary Table S2. OCI-AML3 cells were maintained in DMEM medium (Gibco, Life Technologies, Carlsbad, CA, USA) supplemented with 20% fetal bovine serum (FBS) (Gibco), 1% of penicillin–streptomycin (Gibco) and 4 mM of l-glutamine (Gibco). KG-1, Kasumi-1, NB4, CMK, ME-1 and MOLM-14 cells were maintained in RPMI-1640 medium (Gibco) supplemented with 10% FBS, 1% of penicillin–streptomycin (Gibco) and 2 mM of l-glutamine (Gibco). Cells were grown at 37 °C in 5% CO_2_.

### Cell line differentiation assessment

Macrophage- or granulocytic-like differentiation was induced in OCI-AML3 and ME-1 cell lines with PMA (Sigma-Aldrich, St. Louis, MO, USA) at 100 nM concentration and in NB4 and KG-1 cells with ATRA (Sigma-Aldrich) at 10* μ*M concentration. Cells were seeded at 400 000/ml and were harvested after 24, 48 and 72 h to evaluate cell differentiation.

### Induction culture of AML CD34+ cells

AML CD34+ cells were cultured in StemMACS HSC Expansion medium with StemMACS HSC Expansion Cocktail 1x (Miltenyi Biotec). To induce macrophage-like differentiation 20 ng/ml M-CSF and 1 ng/ml IL-6 (Miltenyi Biotec) were used.

### Flow cytometry

Cytofluorimetric analysis of intracellular Lin28A protein levels was performed after fixation and permeabilization with the IntraCell Kit (Immunostep, Salamanca, Spain) followed by labeling with Lin28A (Cell Signaling Technology, Danvers, MA, USA) or its isotypic control (Cell Signaling Technology) in 11 BM healthy subjects and 9 AML patients. Lin28A protein expression was also evaluated in myeloid, lymphoid and erythroid precursors of CD34+ cells of healthy subjects by using the following fluorochrome-conjugated monoclonal antibodies and their specific isotypic controls: peridin chlorophyll (PerCP) anti-CD45, phycoerythrin (PE) anti-CD33, PE anti-CD19 and PE anti-CD71 (BD Pharmingen). The expression of myeloid-specific antigens CD14, CD11b and CD15 on cell surface was determined by direct immunofluorescent staining with the following fluorochrome-conjugated monoclonal antibodies and their specific isotypic controls: APC anti-CD14, PE anti-CD11b, PE anti-CD11c and PerCP anti-CD15 (BD Pharmingen). For cell-cycle analysis, cells were fixed and permeabilized, and then labeled with PI/RNase staining solution for 30 min. Cells were acquired by FACS Calibur (BD) and analysis was performed using the ModFit LT Software (Verity Software House, Topsham, ME, USA).

### *In vitro* transfection of AML cell lines

Lin28A transfections were performed in OCI-AML3 by using Lipofectamine 2000 (Life Technologies, Carlsbad, CA, USA) in accordance with the manufacturer’s procedure. Transient transfection of *anti-miR-128a* molecule (300 pmol) and negative control (Ambion, Applied Biosistem, Foster City, CA, USA) was accomplished in OCI-AML3 and ME-1 cell lines with Lipofectamine RNAi Max (Life Technologies) in accordance with the manufacturer’s procedure.

### RNA isolation and qRT-PCR for mRNA and miRNA quantification

Mononuclear cells were obtained by Ficoll-Paque gradient centrifugation. Total RNA was extracted using Trizol reagent (Life Technologies) according to the manufacturer’s instructions. Reverse transcription was performed using 1* μ*g of total RNA from each sample by High Capacity cDNA Reverse Transcription Kit (Applied Biosistem, Foster City, CA, USA). qRT-PCR was performed as described previously.^57^ Simultaneous quantification of *ABL1* mRNA was used as a reference for mRNA TaqMan assay data normalization. *miR-128* expression was normalized on *RNU44*. The comparative cycle threshold (Ct) method for relative quantification of mRNA and miRNA expression (User Bulletin No. 2; Applied Biosystems) was used to determine transcript levels.

### Western blotting

Cells were lysed as reported previously.^58^ Total proteins were extracted from AML cell lines. Equal amount of protein extract (60 *μ*g) was transferred to polyvinylidene difluoride membranes (Bio-Rad, Hercules, CA, USA). The membranes were blocked for 1 h with 5% milk (Sigma-Aldrich) at room temperature, and then incubated with primary antibodies directed toward Lin28A (Santa Cruz Biotechnology, Santa Cruz, CA, USA), p21 (Merck Millipore, Billerica, MA, USA) and *β*-actin (Sigma-Aldrich), followed by incubation with horseradish peroxidase-conjugated secondary antibodies (Bio-Rad). Protein bands were visualized and quantified as described previously.^59^

### Lentivirus production and infection

*MiR-128a* expression vector were made by cloning ~60 bp 5′ and 3′ of the pre-miRNA into the multiple cloning site for pLKO.1 (Addgene, Cambridge, MA, USA). Lenti_GFP control and Lenti-miRZip-128a were purchased by System Biosciences (Palo Alto, CA, USA). The virus packaging was performed according to the manufacturer’s instructions. The virus particles (lenti_128a, lenti_GFP control and Lenti-miRZip-128a) were harvested and concentrated using PEG-it Virus Precipitation Solution (System Biosciences). Virus titer was determined in 293TN cells using the global Ultrarapid Lentiviral Titer Kit (System Biosciences). For transduction, AML primary cells and OCI-AML3 were seeded onto 6-well plates at 800 000 cells per ml. Cells were infected with lentiviral stocks at an MOI of 5 in the presence of polybrene. AML primary cells were sorted for the expression of GFP using cell sorter MoFlo Atrios (Beckman Coulter, Brea, CA, USA). OCI-AML3 cells were maintained with puromycin 0.5 *μ*g/ml.

### Colony-forming assay

OCI-AML3 cells infected with pLKO.1_scr or pLKO.1_miR-128a were cultured in 35mm dishes in MethoCult Classic (Stem Cell Technologies, Vancouver, BC, Canada) according to the manufacturer’s instruction. CFU-M were visualized, measured and counted after being cultured in incubator at 37 °C for 14 days.

### May–Grünwald Giemsa staining

OCI-AML3 cells infected with pLKO.1_scr or pLKO.1_miR-128a were harvested at 24, 48 and 72 h after PMA treatment and stained with May–Grünwald for 5 min and Giemsa for 30 min. The cell smears were washed with water, air-dried and observed under optical microscopy (Leica, Wetzlar, Germany).

### Statistical analysis

Results are shown as mean±S.D. or S.E.M. Mann–Whitney *U*-test was used to analyze two group comparisons (protein expression qRT-PCR). Analyses of multiple groups (qRT-PCR of *Lin28A* and *miR-128* in patients and cell lines, *Lin28A* data set analysis) were performed by Dunn's multiple comparisons test after one-way ANOVA with Kruskal–Wallis test. Cytofluorimetric analyses (time course) and qRT-PCR at different time points were carried out by two-way ANOVA followed by *post hoc* multiple comparisons using Sidak’s test. For all tests, a *P*-value <0.05 was taken as statistically significant.

## References

[bib1] Papaemmanuil E, Gerstung M, Bullinger L, Gaidzik VI, Paschka P, Roberts ND et al. Genomic classification and prognosis in acute myeloid leukemia. N Engl J Med 2016; 374: 2209–2221. 27276561 10.1056/NEJMoa1516192 PMC4979995

[bib2] Dohner H, Weisdorf DJ, Bloomfield CD. Acute myeloid leukemia. N Engl J Med 2015; 373: 1136–1152. 26376137 10.1056/NEJMra1406184

[bib3] De Kouchkovsky I, Abdul-Hay M. Acute myeloid leukemia: a comprehensive review and 2016 update. Blood Cancer J 2016; 6: e441. 27367478 10.1038/bcj.2016.50 PMC5030376

[bib4] Arber DA, Orazi A, Hasserjian R, Thiele J, Borowitz MJ, Le Beau MM et al. The 2016 revision to the World Health Organization classification of myeloid neoplasms and acute leukemia. Blood 2016; 127: 2391–2405. 27069254 10.1182/blood-2016-03-643544

[bib5] Fukushima N, Minami Y, Kakiuchi S, Kuwatsuka Y, Hayakawa F, Jamieson C et al. Small-molecule Hedgehog inhibitor attenuates the leukemia-initiation potential of acute myeloid leukemia cells. Cancer Sci 2016; 107: 1422–1429. 27461445 10.1111/cas.13019 PMC5084664

[bib6] Lu FL, Yu CC, Chiu HH, Liu HE, Chen SY, Lin S et al. Sonic hedgehog antagonists induce cell death in acute myeloid leukemia cells with the presence of lipopolysaccharides, tumor necrosis factor-alpha, or interferons. Invest New Drugs 2013; 31: 823–832. 23238608 10.1007/s10637-012-9908-5

[bib7] Chang E, Ganguly S, Rajkhowa T, Gocke CD, Levis M, Konig H. The combination of FLT3 and DNA methyltransferase inhibition is synergistically cytotoxic to FLT3/ITD acute myeloid leukemia cells. Leukemia 2016; 30: 1025–1032. 26686245 10.1038/leu.2015.346 PMC5244408

[bib8] Taskesen E, Staal FJ, Reinders MJ. An integrated approach of gene expression and DNA-methylation profiles of WNT signaling genes uncovers novel prognostic markers in acute myeloid leukemia. BMC Bioinform 2015; 16(Suppl 4): S4. 10.1186/1471-2105-16-S4-S4 PMC4347618 25734857

[bib9] Ye Q, Jiang J, Zhan G, Yan W, Huang L, Hu Y et al. Small molecule activation of NOTCH signaling inhibits acute myeloid leukemia. Scientific Rep 2016; 6: 26510. 10.1038/srep26510 PMC4876435 27211848

[bib10] Takam Kamga P, Bassi G, Cassaro A, Midolo M, Di Trapani M, Gatti A et al. Notch signalling drives bone marrow stromal cell-mediated chemoresistance in acute myeloid leukemia. Oncotarget 2016; 7: 21713–21727. 26967055 10.18632/oncotarget.7964 PMC5008317

[bib11] Gu Y, Masiero M, Banham AH. Notch signaling: its roles and therapeutic potential in hematological malignancies. Oncotarget 2016; 7: 29804–29823. 26934331 10.18632/oncotarget.7772 PMC5045435

[bib12] Jiang S, Baltimore D. RNA-binding protein Lin28 in cancer and immunity. Cancer Lett 2016; 375: 108–113. 26945970 10.1016/j.canlet.2016.02.050

[bib13] Zhou J, Ng SB, Chng WJ. LIN28/LIN28B: an emerging oncogenic driver in cancer stem cells. Int J Biochem Cell Biol 2013; 45: 973–978. 23420006 10.1016/j.biocel.2013.02.006

[bib14] Shyh-Chang N, Daley GQ. Lin28: primal regulator of growth and metabolism in stem cells. Cell Stem Cell 2013; 12: 395–406. 23561442 10.1016/j.stem.2013.03.005 PMC3652335

[bib15] Zhong X, Li N, Liang S, Huang Q, Coukos G, Zhang L. Identification of microRNAs regulating reprogramming factor LIN28 in embryonic stem cells and cancer cells. J Biol Chem 2010; 285: 41961–41971. 20947512 10.1074/jbc.M110.169607 PMC3009922

[bib16] Yu J, Vodyanik MA, Smuga-Otto K, Antosiewicz-Bourget J, Frane JL, Tian S et al. Induced pluripotent stem cell lines derived from human somatic cells. Science 2007; 318: 1917–1920. 18029452 10.1126/science.1151526

[bib17] Glisovic T, Bachorik JL, Yong J, Dreyfuss G. RNA-binding proteins and post-transcriptional gene regulation. FEBS Lett 2008; 582: 1977–1986. 18342629 10.1016/j.febslet.2008.03.004 PMC2858862

[bib18] Wilbert ML, Huelga SC, Kapeli K, Stark TJ, Liang TY, Chen SX et al. LIN28 binds messenger RNAs at GGAGA motifs and regulates splicing factor abundance. Mol Cell 2012; 48: 195–206. 22959275 10.1016/j.molcel.2012.08.004 PMC3483422

[bib19] Hafner M, Max KE, Bandaru P, Morozov P, Gerstberger S, Brown M et al. Identification of mRNAs bound and regulated by human LIN28 proteins and molecular requirements for RNA recognition. RNA 2013; 19: 613–626. 23481595 10.1261/rna.036491.112 PMC3677277

[bib20] Lukong KE, Chang KW, Khandjian EW, Richard S. RNA-binding proteins in human genetic disease. Trends Genet 2008; 24: 416–425. 18597886 10.1016/j.tig.2008.05.004

[bib21] Triboulet R, Pirouz M, Gregory RI. A single Let-7 microRNA bypasses LIN28-mediated repression. Cell Rep 2015; 13: 260–266. 26440890 10.1016/j.celrep.2015.08.086 PMC4607659

[bib22] Li X, Zhang J, Gao L, McClellan S, Finan MA, Butler TW et al. MiR-181 mediates cell differentiation by interrupting the Lin28 and let-7 feedback circuit. Cell Death Differ 2012; 19: 378–386. 21979467 10.1038/cdd.2011.127 PMC3278736

[bib23] Jiang X, Huang H, Li Z, Li Y, Wang X, Gurbuxani S et al. Blockade of miR-150 maturation by MLL-fusion/MYC/LIN-28 is required for MLL-associated leukemia. Cancer Cell 2012; 22: 524–535. 23079661 10.1016/j.ccr.2012.08.028 PMC3480215

[bib24] Bousquet M, Harris MH, Zhou B, Lodish HF. MicroRNA miR-125b causes leukemia. Proc Natl Acad Sci USA 2010; 107: 21558–21563. 21118985 10.1073/pnas.1016611107 PMC3003065

[bib25] O'Connell RM, Chaudhuri AA, Rao DS, Gibson WS, Balazs AB, Baltimore D. MicroRNAs enriched in hematopoietic stem cells differentially regulate long-term hematopoietic output. Proc Natl Acad Sci USA 2010; 107: 14235–14240. 20660734 10.1073/pnas.1009798107 PMC2922591

[bib26] Chaudhuri AA, So AY, Mehta A, Minisandram A, Sinha N, Jonsson VD et al. Oncomir miR-125b regulates hematopoiesis by targeting the gene Lin28A. Proc Natl Acad Sci USA 2012; 109: 4233–4238. 22366319 10.1073/pnas.1200677109 PMC3306721

[bib27] Yuan J, Nguyen CK, Liu X, Kanellopoulou C, Muljo SA. Lin28b reprograms adult bone marrow hematopoietic progenitors to mediate fetal-like lymphopoiesis. Science 2012; 335: 1195–1200. 22345399 10.1126/science.1216557 PMC3471381

[bib28] Qian P, Banerjee A, Wu ZS, Zhang X, Wang H, Pandey V et al. Loss of SNAIL regulated miR-128-2 on chromosome 3p22.3 targets multiple stem cell factors to promote transformation of mammary epithelial cells. Cancer Res 2012; 72: 6036–6050. 23019226 10.1158/0008-5472.CAN-12-1507

[bib29] Georgantas RW III, Hildreth R, Morisot S, Alder J, Liu CG, Heimfeld S et al. CD34+ hematopoietic stem-progenitor cell microRNA expression and function: a circuit diagram of differentiation control. Proc Natl Acad Sci USA 2007; 104: 2750–2755. 17293455 10.1073/pnas.0610983104 PMC1796783

[bib30] Shim J, Nam JW. The expression and functional roles of microRNAs in stem cell differentiation. BMB Rep 2016; 49: 3–10. 26497582 10.5483/BMBRep.2016.49.1.217 PMC4914210

[bib31] Seca H, Lima RT, Almeida GM, Sobrinho-Simoes M, Bergantim R, Guimaraes JE et al. Effect of miR-128 in DNA damage of HL-60 acute myeloid leukemia cells. Curr Pharm Biotechnol 2014; 15: 492–502. 24846063 10.2174/1389201015666140519122524

[bib32] Mi S, Lu J, Sun M, Li Z, Zhang H, Neilly MB et al. MicroRNA expression signatures accurately discriminate acute lymphoblastic leukemia from acute myeloid leukemia. Proc Natl Acad Sci USA 2007; 104: 19971–19976. 18056805 10.1073/pnas.0709313104 PMC2148407

[bib33] Seca H, Almeida GM, Guimaraes JE, Vasconcelos MH. miR signatures and the role of miRs in acute myeloid leukaemia. Eur J Cancer 2010; 46: 1520–1527. 20413298 10.1016/j.ejca.2010.03.031

[bib34] Pham TH, Benner C, Lichtinger M, Schwarzfischer L, Hu Y, Andreesen R et al. Dynamic epigenetic enhancer signatures reveal key transcription factors associated with monocytic differentiation states. Blood 2012; 119: e161–e171. 22550342 10.1182/blood-2012-01-402453

[bib35] Noiret M, Hardy S, Audic Y. zfp36 expression delineates both myeloid cells and cells localized to the fusing neural folds in *Xenopus tropicalis*. Int J Dev Biol 2014; 58: 751–755. 26154316 10.1387/ijdb.140264ya

[bib36] Chen MT, Dong L, Zhang XH, Yin XL, Ning HM, Shen C et al. ZFP36L1 promotes monocyte/macrophage differentiation by repressing CDK6. Scientific Rep 2015; 5: 16229. 10.1038/srep16229 PMC4635361 26542173

[bib37] Montero-Melendez T, Dalli J, Perretti M. Gene expression signature-based approach identifies a pro-resolving mechanism of action for histone deacetylase inhibitors. Cell Death Differ 2013; 20: 567–575. 23222458 10.1038/cdd.2012.154 PMC3595482

[bib38] Matsuo Y, MacLeod RA, Uphoff CC, Drexler HG, Nishizaki C, Katayama Y et al. Two acute monocytic leukemia (AML-M5a) cell lines (MOLM-13 and MOLM-14) with interclonal phenotypic heterogeneity showing MLL-AF9 fusion resulting from an occult chromosome insertion, ins(11;9)(q23;p22p23). Leukemia 1997; 11: 1469–1477. 9305600 10.1038/sj.leu.2400768

[bib39] Ivey A, Hills RK, Simpson MA, Jovanovic JV, Gilkes A, Grech A et al. Assessment of minimal residual disease in standard-risk AML. N Engl J Med 2016; 374: 422–433. 26789727 10.1056/NEJMoa1507471

[bib40] Kueh HY, Champhekar A, Nutt SL, Elowitz MB, Rothenberg EV. Positive feedback between PU.1 and the cell cycle controls myeloid differentiation. Science 2013; 341: 670–673. 23868921 10.1126/science.1240831 PMC3913367

[bib41] Carter JH, Tourtellotte WG. Early growth response transcriptional regulators are dispensable for macrophage differentiation. J Immunol 2007; 178: 3038–3047. 17312150 10.4049/jimmunol.178.5.3038

[bib42] Sugimoto MA, Vago JP, Teixeira MM, Sousa LP. Annexin A1 and the resolution of inflammation: modulation of neutrophil recruitment, apoptosis, and clearance. J Immunol Res 2016; 2016: 8239258. 26885535 10.1155/2016/8239258 PMC4738713

[bib43] Kim SH, Kim MO, Cho YY, Yao K, Kim DJ, Jeong CH et al. ERK1 phosphorylates Nanog to regulate protein stability and stem cell self-renewal. Stem Cell Res 2014; 13: 1–11. 24793005 10.1016/j.scr.2014.04.001

[bib44] Forget MA, Voorhees JL, Cole SL, Dakhlallah D, Patterson IL, Gross AC et al. Macrophage colony-stimulating factor augments Tie2-expressing monocyte differentiation, angiogenic function, and recruitment in a mouse model of breast cancer. PLoS ONE 2014; 9: e98623. 24892425 10.1371/journal.pone.0098623 PMC4043882

[bib45] Seipel K, Marques MT, Bozzini MA, Meinken C, Mueller BU, Pabst T. Inactivation of the p53-KLF4-CEBPA axis in acute myeloid leukemia. Clin Cancer Res 2016; 22: 746–756. 26408402 10.1158/1078-0432.CCR-15-1054

[bib46] Saudy NS, Fawzy IM, Azmy E, Goda EF, Eneen A, Abdul Salam EM. BMI1 gene expression in myeloid leukemias and its impact on prognosis. Blood Cell Mol Dis 2014; 53: 194–198. 10.1016/j.bcmd.2014.07.002 25084695

[bib47] Kakiuchi S, Minami Y, Miyata Y, Mizutani Y, Goto H, Kawamoto S et al. NANOG expression as a responsive biomarker during treatment with Hedgehog signal inhibitor in acute myeloid leukemia. Int J Mol Sci 2017; 18: 486. 10.3390/ijms18030486 PMC5372502 28245563

[bib48] Garofalo M, Croce CM. Role of microRNAs in maintaining cancer stem cells. Adv Drug Deliv Rev 2015; 81: 53–61. 25446141 10.1016/j.addr.2014.11.014 PMC4445133

[bib49] Yuan Y, Kasar S, Underbayev C, Prakash S, Raveche E. MicroRNAs in acute myeloid leukemia and other blood disorders. Leuk Res Treat 2012; 2012: 603830. 10.1155/2012/603830 PMC3505936 23259069

[bib50] Marcucci G, Mrozek K, Radmacher MD, Garzon R, Bloomfield CD. The prognostic and functional role of microRNAs in acute myeloid leukemia. Blood 2011; 117: 1121–1129. 21045193 10.1182/blood-2010-09-191312 PMC3056468

[bib51] Yendamuri S, Calin GA. The role of microRNA in human leukemia: a review. Leukemia 2009; 23: 1257–1263. 19148134 10.1038/leu.2008.382

[bib52] Gangaraju VK, Lin H. MicroRNAs: key regulators of stem cells. Nat Rev Mol Cell Biol 2009; 10: 116–125. 19165214 10.1038/nrm2621 PMC4118578

[bib53] Vargas Romero P, Cialfi S, Palermo R, De Blasio C, Checquolo S, Bellavia D et al. The deregulated expression of miR-125b in acute myeloid leukemia is dependent on the transcription factor C/EBPalpha. Leukemia 2015; 29: 2442–2445. 25982911 10.1038/leu.2015.117 PMC4675867

[bib54] Su R, Lin HS, Zhang XH, Yin XL, Ning HM, Liu B et al. MiR-181 family: regulators of myeloid differentiation and acute myeloid leukemia as well as potential therapeutic targets. Oncogene 2015; 34: 3226–3239. 25174404 10.1038/onc.2014.274

[bib55] Caivano A, La Rocca F, Simeon V, Girasole M, Dinarelli S, Laurenzana I et al. MicroRNA-155 in serum-derived extracellular vesicles as a potential biomarker for hematologic malignancies – a short report. Cell Oncol 2016; 40: 97–103. 10.1007/s13402-016-0300-x PMC13001561 27761889

[bib56] Ferrara F, Di Noto R, Viola A, Russo C, Boccuni P, Costantini S et al. Complete remission in acute myeloid leukaemia with t(8;21) following treatment with G-CSF: flow cytometric analysis of *in vivo* and *in vitro* effects on cell maturation. Br J Haematol 1999; 106: 520–523. 10460615 10.1046/j.1365-2141.1999.01580.x

[bib57] De Luca L, Trino S, Laurenzana I, Simeon V, Calice G, Raimondo S et al. MiRNAs and piRNAs from bone marrow mesenchymal stem cell extracellular vesicles induce cell survival and inhibit cell differentiation of cord blood hematopoietic stem cells: a new insight in transplantation. Oncotarget 2016; 7: 6676–6692. 26760763 10.18632/oncotarget.6791 PMC4872742

[bib58] Trino S, Iacobucci I, Erriquez D, Laurenzana I, De Luca L, Ferrari A et al. Targeting the p53-MDM2 interaction by the small-molecule MDM2 antagonist Nutlin-3a: a new challenged target therapy in adult Philadelphia positive acute lymphoblastic leukemia patients. Oncotarget 2016; 7: 12951–12961. 26887044 10.18632/oncotarget.7339 PMC4914334

[bib59] Laurenzana I, Caivano A, Trino S, De Luca L, La Rocca F, Simeon V et al. A pyrazolo[3,4-*d*]pyrimidine compound inhibits Fyn phosphorylation and induces apoptosis in natural killer cell leukemia. Oncotarget 2016; 7: 65171–65184. 27566560 10.18632/oncotarget.11496 PMC5323146

