# Supplementary material for: Knockdown of miR-128a induces Lin28a expression and reverts myeloid differentiation blockage in acute myeloid leukemia
Source: Cell Death Dis. 2017 Jun 1;8(6):e2849–. doi: 10.1038/cddis.2017.253 (PMC5520910; doi:10.1038/cddis.2017.253)
Supplement: Supplementary Figure 1 [file cddis2017253x1.doc]

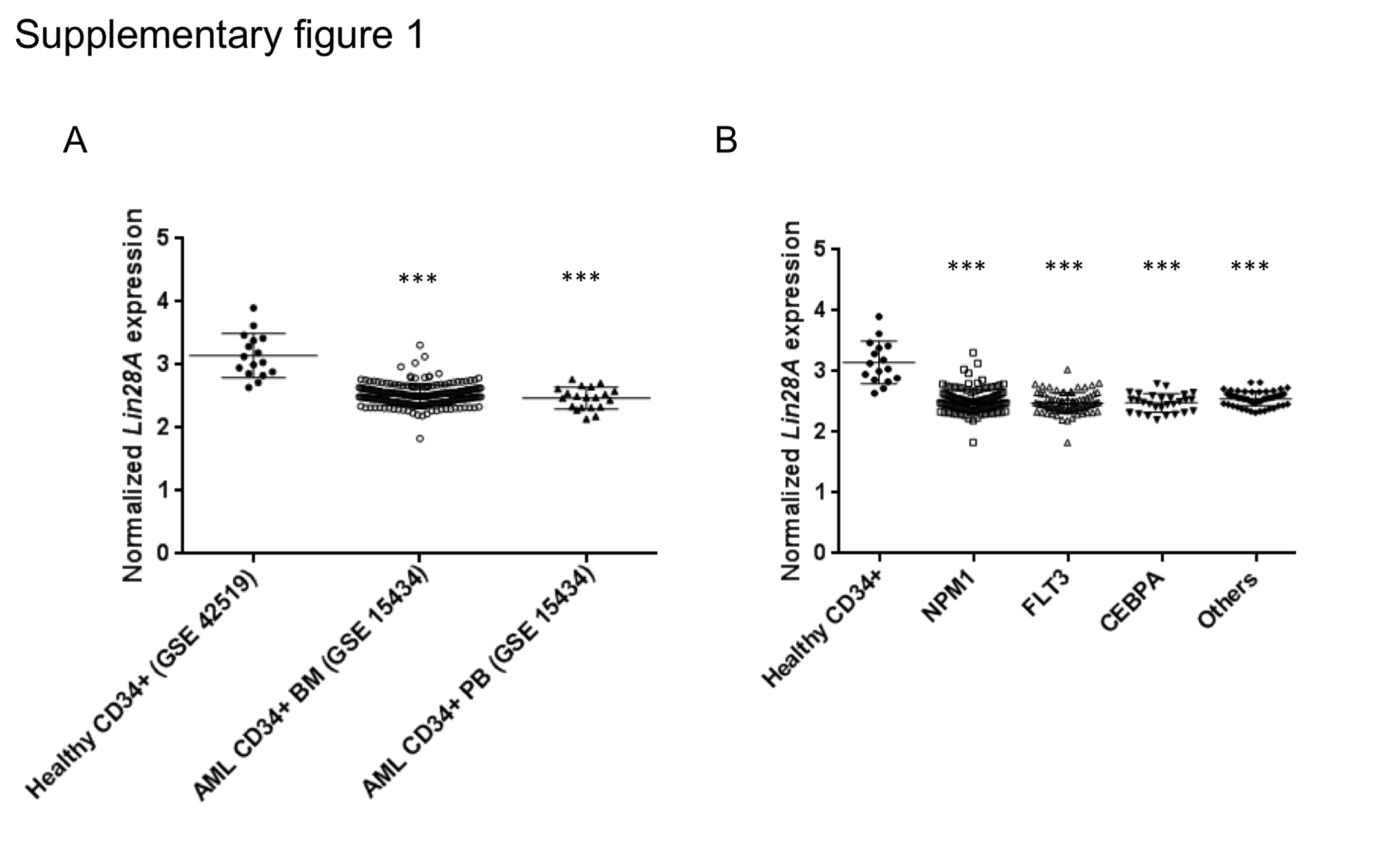


Supplementary Figure 1: **(A)** *Lin28A* expression level in AML patients (230 sample from BM and 21 from PB) (GSE 15434) compared with 16 CD34+ isolated cells from healthy subjects (GSE 42519). **(B)** *Lin28A* expression level in AML patients (GSE 15434) stratified for different mutations 128 NPM1 (n=128), FLT3 (n=81), CEBPA (n=28), Others (n=56) compared with CD34+ isolated cells from healthy subjects healthy subjects (n=16) (GSE 42519).

Statistically significant analysis are indicated by asterisks: ***p value<0.001.
