# Supplementary material for: Knockdown of miR-128a induces Lin28a expression and reverts myeloid differentiation blockage in acute myeloid leukemia
Source: Cell Death Dis. 2017 Jun 1;8(6):e2849–. doi: 10.1038/cddis.2017.253 (PMC5520910; doi:10.1038/cddis.2017.253)
Supplement: Supplementary Figure 2 [file cddis2017253x2.doc]

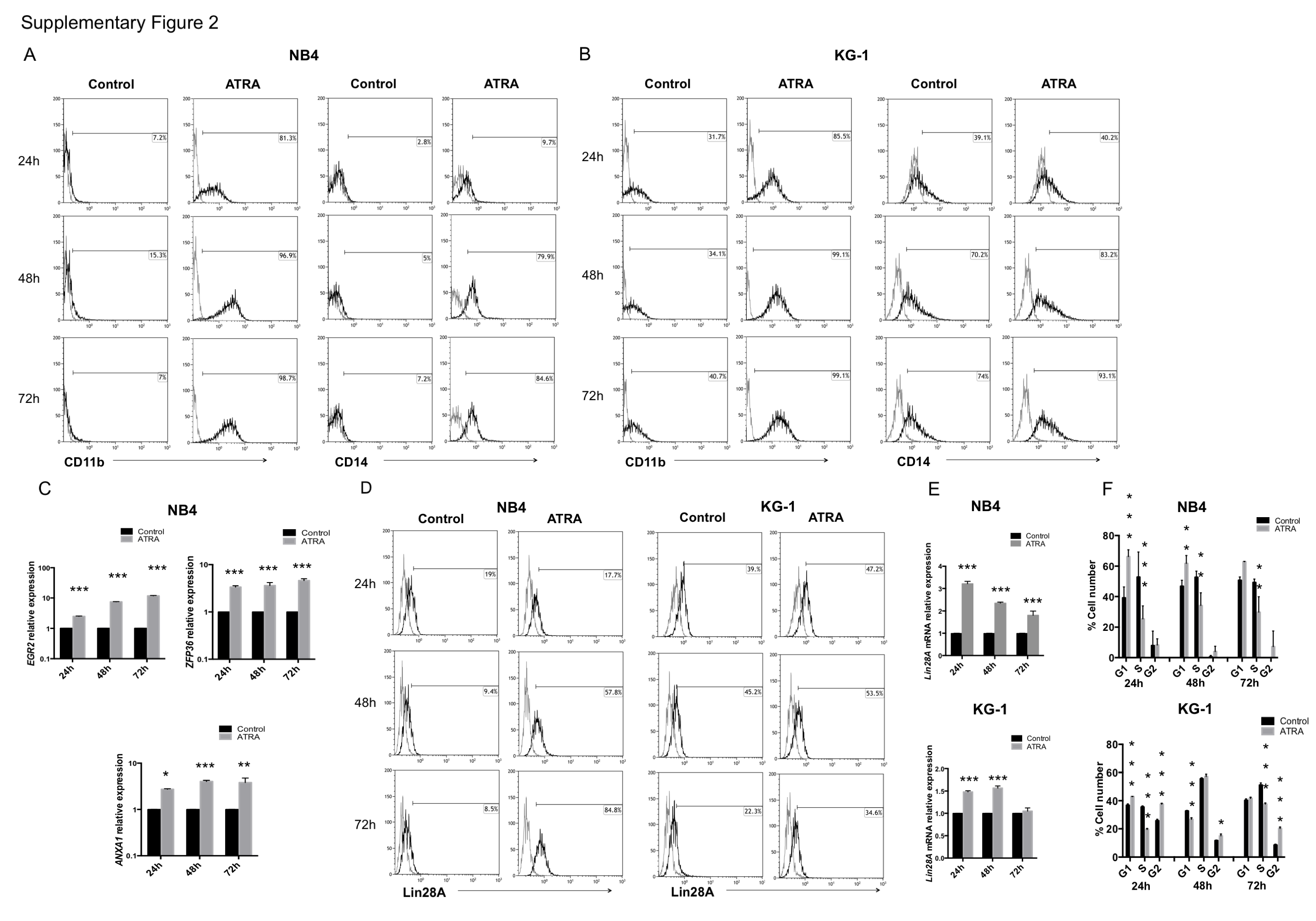


Supplementary Figure 2: (A-B) Percentage of CD11b and CD14 positive cells in NB4 (A) and CD11b and CD15 positive cells in KG-1 (B) after 24h, 48h and 72h of treatment with ATRA, by cytofluorimetric analysis. (C) qRT-PCR of *EGR2, ZFP36* and *ANXA1* in NB4 cells after 24h, 48h and 72h of treatment with ATRA. (D) Percentage of Lin28A positive cells in NB4 and KG-1 cells after 24h, 48h and 72h of treatment with ATRA, by cytofluorimetric analysis. (E) qRT-PCR of *Lin28A* in NB4 and KG-1 cells after 24h, 48h and 72h of treatment with ATRA. (F) Cell cycle analysis in NB4 and KG-1 cells after 24h, 48h and 72h of treatment with ATRA.

The bar-graphs represented mean + SD from three independent experiments.

Statistically significant analyses are indicated by asterisks: * p<0.05, ** p<0.01, *** p<0.001.
