# Supplementary material for: Knockdown of miR-128a induces Lin28a expression and reverts myeloid differentiation blockage in acute myeloid leukemia
Source: Cell Death Dis. 2017 Jun 1;8(6):e2849–. doi: 10.1038/cddis.2017.253 (PMC5520910; doi:10.1038/cddis.2017.253)
Supplement: Supplementary Figure 3 [file cddis2017253x3.doc]

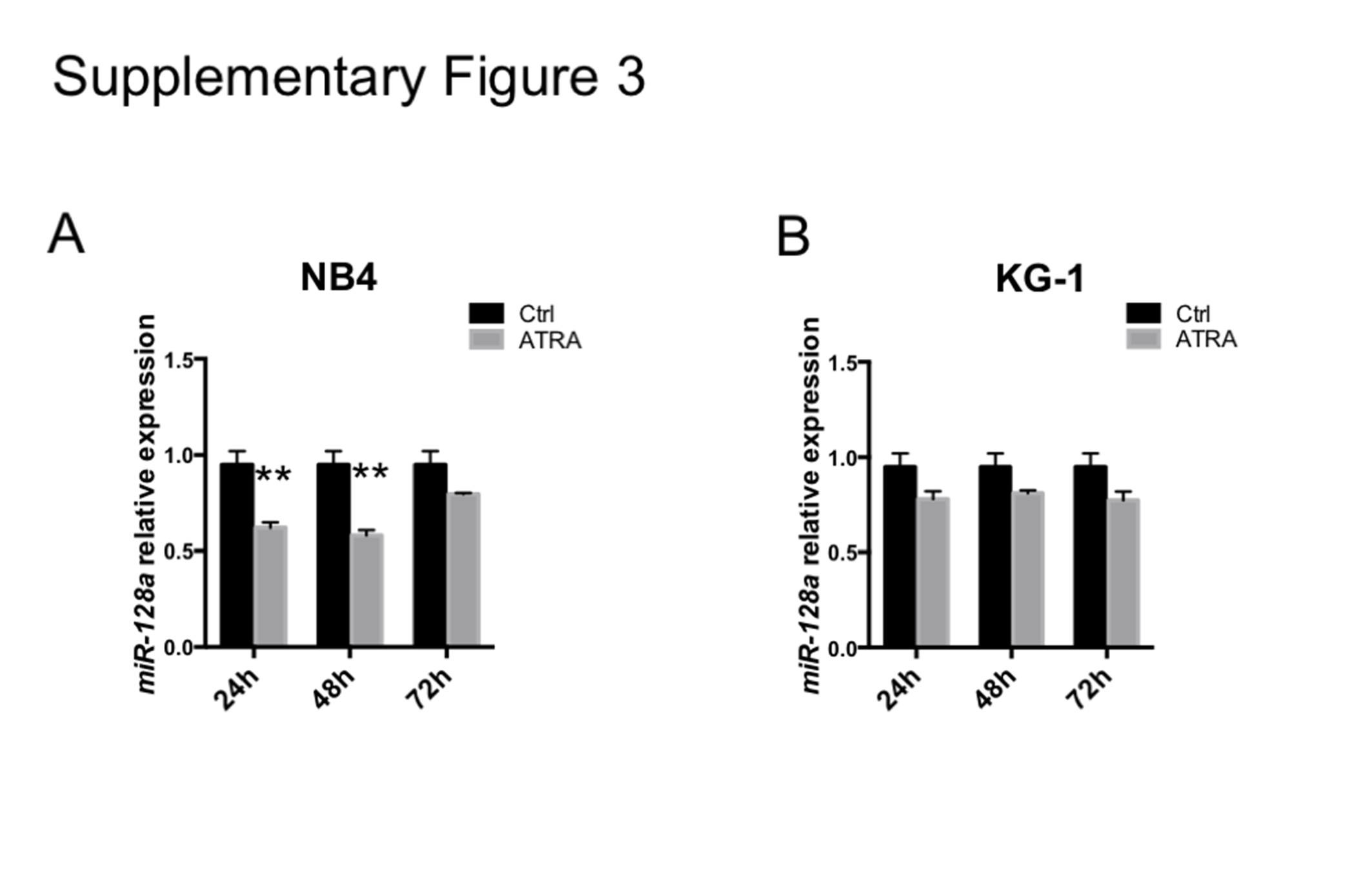


**Supplementary Figure 3**: **(A-B)** qRT-PCR of *miR-128a* in NB4 (A) and KG-1 (B) after 24h, 48h and 72h of treatment with ATRA. The bar-graphs represented mean + SD from three independent experiments. Statistically significant analyses are indicated by asterisks: ** p<0.01
