# Supplementary material for: Knockdown of miR-128a induces Lin28a expression and reverts myeloid differentiation blockage in acute myeloid leukemia
Source: Cell Death Dis. 2017 Jun 1;8(6):e2849–. doi: 10.1038/cddis.2017.253 (PMC5520910; doi:10.1038/cddis.2017.253)
Supplement: Supplementary Figure 4 [file cddis2017253x4.doc]

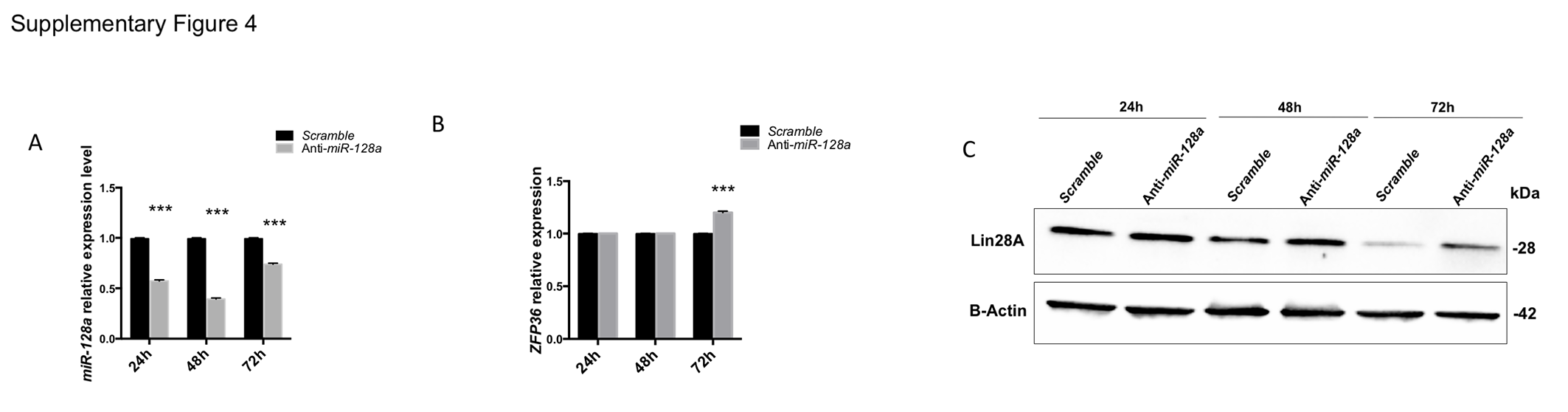


**Supplementary Figure 4: (A-B)** qRT-PCR of *miR-128a* (A) and *ZFP36* (B) in ME-1 after 24h, 48h and 72h of scramble or anti-miR128a transfection. **(C)** WB analysis of Lin28A and β-Actin in ME-1 after 24h, 48h and 72h of transfection with scramble or anti-miR128a. The bar-graphs represented mean + SD from three independent experiments.

Statistically significant analyses are indicated by asterisks: *** p<0.001.
