# Supplementary material for: Knockdown of miR-128a induces Lin28a expression and reverts myeloid differentiation blockage in acute myeloid leukemia
Source: Cell Death Dis. 2017 Jun 1;8(6):e2849–. doi: 10.1038/cddis.2017.253 (PMC5520910; doi:10.1038/cddis.2017.253)
Supplement: Supplementary Figure 5 [file cddis2017253x5.doc]

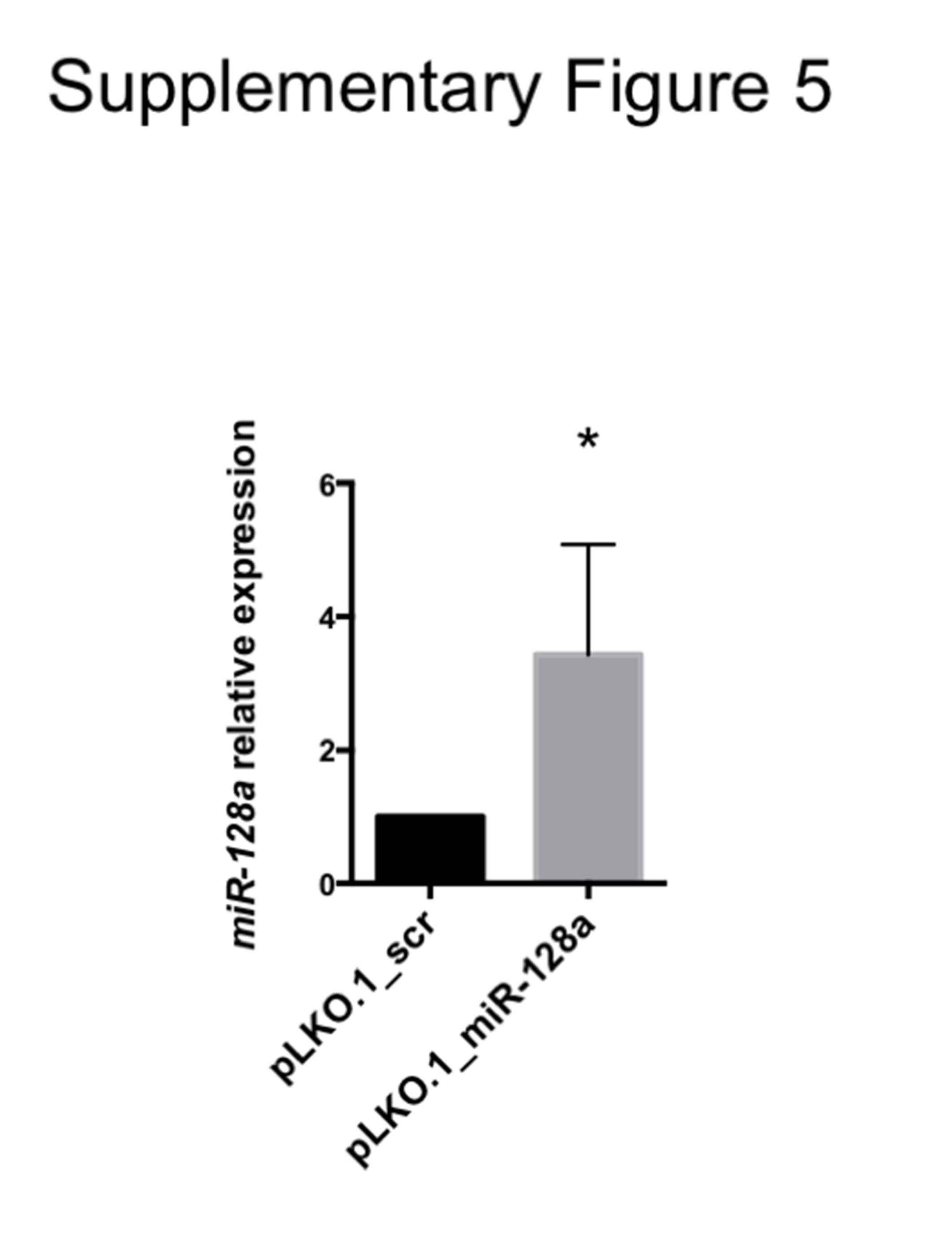


**Supplementary Figure 5**: qRT-PCR of *miR-128a* in OCI-AML3 after infection with pLKO.1_scr or pLKO.1_miR-128a. The bar-graphs represented mean + SD from three independent experiments.

Statistically significant analyses are indicated by asterisks: *p<0.05.
