# Supplementary material for: Knockdown of miR-128a induces Lin28a expression and reverts myeloid differentiation blockage in acute myeloid leukemia
Source: Cell Death Dis. 2017 Jun 1;8(6):e2849–. doi: 10.1038/cddis.2017.253 (PMC5520910; doi:10.1038/cddis.2017.253)
Supplement: Supplementary Table 1 [file cddis2017253x6.pdf]

**Supplementary table 1:** Clinical-biological features of AML patients

| UPN | % blasts in BM | WHO Classification | Mutation status | Cytogenetics | *Lin28A* expression | *miR-128a* expression |
|---|---|---|---|---|---|---|
| 1 | 90 | APL | PML/RARα | 46,XY,t(15;17)(q24;q21),der(3)t(3;?)or add(3)(q?)[20] | 0.000103721 | 0.181 |
| 2 | 90 | Acute myelomonocytic leukemia | WT1(+) FLT3(+) | Normal | 0.000468 | 0.025 |
| 3 | 75 | AML with maturation | WT1(+) FLT3(+) | Complex karyptype | 0.000114 | 0.027 |
| 4 | 97 | Not specified | WT1(+) | 46,X?,inv(16)(p13q22) | 0.003940 | 0.008 |
| 5 | 90 | Not specified | WT1(+) NPM1(+) FLT3(+) | Not available | 0.0477 | 0.033146492 |
| 6 | 100 | Not specified | WT1(+) FLT3(+) MLL(+) | Not available | 0.002420 | |
| 7 | 85 | Not specified | WT1(+) NPM1(+) | Not available | 0.002230 | 0.047530586 |
| 8 | 90 | Not specified | WT1(+) FLT3(+) | Not available | | 0.014477938 |
| 9 | 100 | APL | WT1(+) PML/RARα | 46,XY,t(15;17)(q24;q21) | 0.003880 | 0.060162153 |
| 10 | 100 | APL | WT1(+) PML/RARα FLT3(+) | 46,XX,t(15;17)(q24;q21) | 0.003360 | 0.129408115 |
| 11 | 85 | | | | 0.000324 | 0.022405551 |
| 12 | 80 | Acute myelomonocytic leukemia | WT1(+) NPM1(+) FLT3(+) | Not available | 0.001350 | 0.010273 |
| 13 | 98 | | | | | 0.01132 |
| 14 | 60 | Acute monoblastic/monocytic leukemia | WT1(+) | Not available | 0.001540 | 0.014 |
| 15 | 97 | AML with maturation | WT1(+) NPM1(+) | Not available | 0.001630 | 0.013 |
| 16 | 90 | Secondary AML | | Not available | 0.001790 | 0.01589812 |
| 17 | 90 | | | Not available | 0.014400 | 0.008912217 |
| 18 | 90 | AML with maturation | WT1(+) | Not available | 0.009300 | 0.018198962 |
| 19 | 70 | Secondary AML | WT1(+) | Complex karyotype | 0.000146 | 0.081899588 |
| 20 | 81 | AML without maturation | WT1(+) NPM1(+) FLT3(+) | Not available | 0.000459 | |
| 21 | 80 | AML without maturation | WT1(+) | 46,XY,t(9;11) | 0.00248 | 0.01 |
| 22 | 98 | AML with minimal | NPM1(+) | Normal | 0.000564 | |

| | | | | | | |
|---|---|---|---|---|---|---|
| | | differentiation | | | | |
| **23** | 82 | AML without maturation | | Normal | 0.0015 | 0.011 |
| **24** | 90 | Acute myelomonocytic leukemia | | Not available | 0.0295 | 0.018000 |
| **25** | 95 | Acute monoblastic/monocytic leukemia | WT1(+) | 46,XX,inv(16)(p13q22),t(X;5)(q?;q?) | 0.011 | 0.046000 |
| **26** | 90 | AML without maturation | | Normal | 0.0008 | |
| **27** | 70-80 | Acute myelomonocytic leukemia | WT1(+) | Not available | 0.0004 | |
| **28** | 60-70 | Acute myelomonocytic leukemia | WT1(+) | Normal | 0.000429 | 0.018 |
| **29** | 80 | Acute myelomonocytic leukemia | | Not available | 0.000614 | 0.01 |
| **30** | 90 | AML with minimal differentiation | WT1(+) | Not available | 0.000573 | 0.01 |
| **31** | 80 | AML without maturation | NPM1(+) | Normal | 0.000533 | 0.012 |
| **32** | 95 | Acute myelomonocytic leukemia | FLT3(+) | Not available | 0.00122 | 0.021 |
| **33** | 70 | AML without maturation | WT1(+) | 45,XY,-7[?] | 0.000887 | 0.014 |
| **34** | 70 | Acute myelomonocytic leukemia | WT1(+) | Complex karyotype | 0.00139 | 0.013 |
| **35** | 80 | AML without maturation | NPM1(+) | | 0.000573 | 0.011 |
| **36** | 90 | Acute myelomonocytic leukemia | WT1(+) | Not available | 0.000188 | 0.014477938 |
| **37** | 69 | Secondary AML | WT1(+) | Not available | 0.000821188 | 0.017579039 |
| **38** | 90 | Acute myelomonocytic leukemia | WT1(+) | Complex karyotype | 0.000143168 | 0.01 |
| **39** | 76 | AML without maturation | WT1(+); NPM1(+) | Normal | 0.010380358 | 0.022 |
| **40** | 65 | AML without maturation | WT1(+) | 46,XY,del(9)(q21)[20] | 0.000171441 | 0.068 |

UPN, unique patient number; BM, bone marrow; APL, acute promyelocytic leukemia; AML, acute myeloid leukemia.
