# Supplementary material for: Knockdown of miR-128a induces Lin28a expression and reverts myeloid differentiation blockage in acute myeloid leukemia
Source: Cell Death Dis. 2017 Jun 1;8(6):e2849–. doi: 10.1038/cddis.2017.253 (PMC5520910; doi:10.1038/cddis.2017.253)
Supplement: Supplementary Table 2 [file cddis2017253x7.pdf]

Supplementary table 2: Clinical-biological features of AML cell lines

AML cell line	Classification	Karyotype	Genetic alterations
**OCI-AML3**	Acute myelomonocytic leukemia	Hyperdiploid; 48(45-50)<2n>X/XY,+1,+5,+8, der(1)t(1;18)(p11;q11),i(5p),del(13)(q13q21), dup(17)(q21q25); sideline with r(Y)x1-2 - hemizygous for RB1	NPM1(+); DNMT3A(+)
**KG-1**	AML with minimal differentiation	Near-diploid; 46,XY,-5,-7,-8,-12,-17,+mar/46,XY,idem,+5mar	
**Kasumi-1**	AML with maturation	t(8;21)(q22;q22)	*RUNX1(AML1)/CBFA2T1(ETO)*
**NB-4**	APL	Human hypertriploid karyotype with 3% polyploidy; 78(71-81)<3n>XX,-X,+2,+6,+7,+7,+11,+12,+13,+14,+17,-19,+20,+4mar, der(8)t(8;?)(q24;?),der(11)t(11;?)(?->::11p15->11q22.1::11q13->22.1:),der(12)t(12;?)(p11;?),14p+, t(15;17)(q22;q11-12.1),der(19)t(10;19)(q21.1;p13.3)x2	*PML/RARα*
**CMK**	Acute megakaryoblastic leukemia	Human flat-moded hypotetraploid karyotype with 8% polyploidy; 85-90<4n>XY,-X,-Y,-2,-3,+5,-6,-6,-8,+11,-15,-15,+16,-17,-19,+21,+22,+7-11mar, add(1)(q31),add(1)(p36),add(3)(q11),del(3)(p14)x2-3,add(5)(q11),add(5)(q13),dup(8)(q11q21),add(8)(q13-21),del(8)(q11),del(9)(p21)x2,add(9)(q11)x2,del(10)(q22q24),der(11;17)(q10;q10),der(11)dup(11)(p13p15)t(5;11)(q11;p15)x1-2,del(11)(q23),add(12)(p13)x2, add(17)(p1?),add(18)(q23)x2-3, add(19)(p13),der(20)t(1;20)(q2?5;q1?2)x2,add(22)(q13)	
**ME-1**	Acute myelomonocytic leukemia	Human hyperdiploid karyotype with 9% polyploidy; 48(45-48)<2n>XY,+8,+20,der(2)t(2;11)(q31;q13), der(9)t(9;18)(q12.2;q11),der(16)t(11;16)(q13;q22)inv(16)(p13q22),der(17)t(2;17)(p25;p12); sdl lacking trisomy 20; carries inversion/translocation effecting rearrangement of MYH11 and CBFB	*CBFB/MYH11*
**MOLM-14**	Acute monoblastic/monocytic leukemia	Human hyperdiploid karyotype; 49(46-50)<2n>XY,+6,+8,+13,der(2)t(1;2)(q31;q35),ins(11;9)(q23;p22p23),del(14)(q23q32.3),del(16)(q11.2q13.1); sdl without der(2) but with tetrasomy 8 and/or i(8)(q10); carries insertional variant of t(9;11) recurrent in AML, effecting rearrangement of MLL (KMT2A) with MLLT3 (AF9); ins(11;9) plus trisomies 8 and 13 also present in sister cell line MOLM-13 which it closely resembles	*FLT3-ITD*; *CBL* (+)

AML, acute myeloid leukemia; APL, acute promyelocytic leukemia.
